# Human Papillomavirus-Related Multiphenotypic Sinonasal Carcinoma—An Even Broader Tumor Entity?

**DOI:** 10.3390/v13091861

**Published:** 2021-09-17

**Authors:** Mark Zupancic, Anders Näsman

**Affiliations:** Department of Oncology-Pathology, Karolinska Institutet, Bioclinicum J6:20, Karolinska University Hospital, 17164 Stockholm, Sweden; Mark.Zupancic@ki.se

**Keywords:** human papillomavirus, human papillomavirus-related multiphenotypic sinonasal carcinoma, head and neck cancer, basaloid squamous cell carcinoma

## Abstract

Human papillomavirus (HPV)-related multiphenotypic sinonasal carcinoma (HMSC) is a recently defined tumor subtype with apparent favorable clinical outcome despite aggressive histomorphology. However, in recent years, additional numbers of cases, with more variable features and at locations outside the sinonasal region, have complicated the definition of HMSC. Here, we have performed a systematic review of all cases described so far in order to accumulate more knowledge. We identified 127 articles published between 2013 and 2021, of which 21 presented unique cases. In total, 79 unique patient cases were identified and their clinical and micromorphological nature are herein summarized. In our opinion, better clinical follow-up data and a more detailed tumor characterization are preferably needed before HMSC can finally be justified as its own tumor entity.

## 1. Introduction

Since the first case report in 2013 by Bishop and colleagues, in which six patients with the potentially new entity at that time called human papillomavirus (HPV)-related carcinoma with adenoid cystic carcinoma-like features was described [1], additional reports have been published from various parts of the world. Although there have been a few early attempts to summarize all of the published cases of HPV-related multiphenotypic sinonasal carcinoma (HMSC), no systematic review has, to our knowledge, been conducted so far [2]. In addition, HMSC was initially described as a relatively uniform tumor type in terms of localization, clinical behavior, HPV subtype and histomorphology. However, recent case reports have suggested that the clinical and micromorphological spectrum might be broader than initially reported. Therefore, we aimed to review the current knowledge about HMSC by performing a systematic review of, to our knowledge, all cases published so far and summarize their clinical and histopathological characteristics.

## 2. Materials and Methods

**Figure 1:**
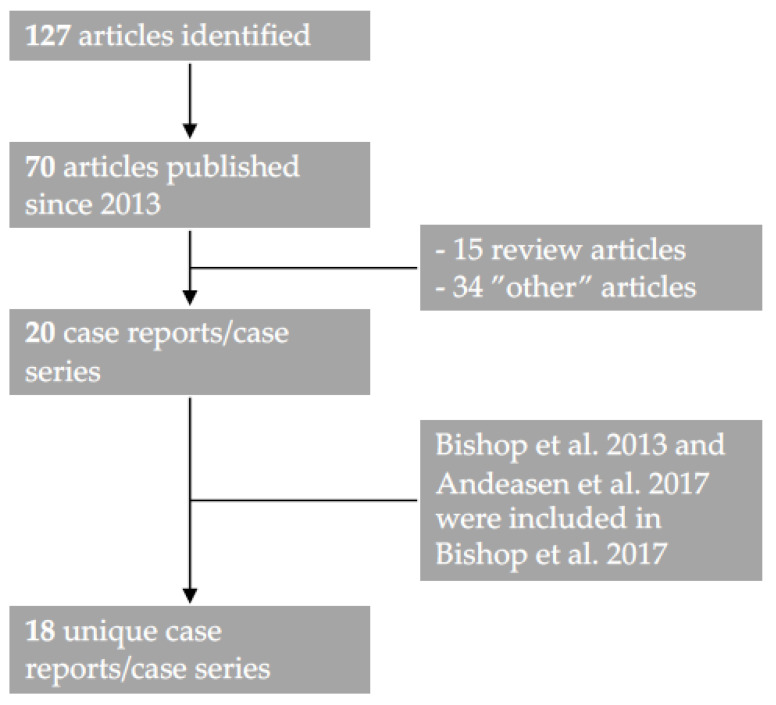
Flow diagram with the selection process of articles. Initially, 127 articles were identified using the search terms ““multiphenotypic” OR “multiphenotypic sinonasal” OR “adenoid cystic like””, of which 70 manuscripts were published since the first case report in 2013. After reviewing all abstracts, 20 reports were identified, excluding 15 review articles without additional cases reported and 34 articles outside the topic (“other” articles). Bishop et al. 2017 [3] summarized and included all data from Bishop et al. 2013 [1] and Andreasen 2017 [4] and added 35 new unique cases. Therefore, 18 unique case series were identified.

We adhered to the PRISMA guidelines and searched PubMed, using the search terms ““multiphenotypic” OR “multiphenotypic sinonasal” OR “adenoid cystic like”” in May 2021 and found 127 unique articles, of which 69 articles were published after the first case report in 2013 (*n* = 70). Subsequently, one researcher (AN) reviewed the abstracts of all 70 articles and found 21 articles that met the inclusion criteria (a patient case or a series of patients diagnosed with “HPV-related carcinoma with adenoid cystic carcinoma-like features”/HMSC, written in English) (Figure 1). Full-text versions of all manuscripts were available in all cases. Three articles had overlapping patient cases [1,3,4], with all patients covered in Bishop et al. 2017 [3].

Patient and tumor data (age, sex, tumor localization and size, treatment, outcome and follow-up time in months after treatment), pathological parameters (morphology, immunohistochemistry staining (IHC) data) and HPV status (p16, HPV DNA PCR, HPV DNA ISH and HPV RNA ISH status) were collected by two researchers independently (AN, MZ). The outcomes were classified as “No evidence of disease” (NED), “Local relapse” (LR), “Distant metastasis” (DM) or “Dead of disease“ (DOD) and patients were classified according to their first reported event. Event-free survival was calculated and was defined as the time from first diagnosis (months) until the first reported event (LR, DM, DOD). Patients with NED were censored at the last day of follow-up. All statistics were performed in SPSS (IBM Corp. Released 2017. IBM SPSS Statistics for Macintosh, Version 25.0. Armonk, NY, USA: IBM Corp.).

## 3. Results

### 3.1. Patients and Clinical Characteristics

In total, 21 articles were identified, including 79 unique patients diagnosed with HMSC (Table 1), and of these patients, 39 (51%) were women, 38 (49%) men and 1 case was not specified. Seventy-eight patients had a primary tumor within the head and neck region, while one case was reported outside this region (breast). The mean and median ages were 53.7 and 53 years, respectively, for the total cohort (53.6 and 53 years, respectively, in patients with head and neck tumors).

When focusing on patients with head and neck tumors only, the most common primary site was the nasal cavity but, notably, the primary site was not restricted to the sinonasal region. Tumor size was specified in 57 cases and the total mean size was 3.5 cm. The presence of regional metastasis was only reported in one case (case 78; primary tonsil tumor). For cases in which treatment was specified (*n* = 61), the most common treatment modality was surgery only (*n* = 34), followed by surgery and radiotherapy (RT) (*n* = 21); surgery, RT and chemotherapy (CT) (*n* = 4); RT alone (*n* = 1); and CT alone (*n* = 1).

**Figure 2:**
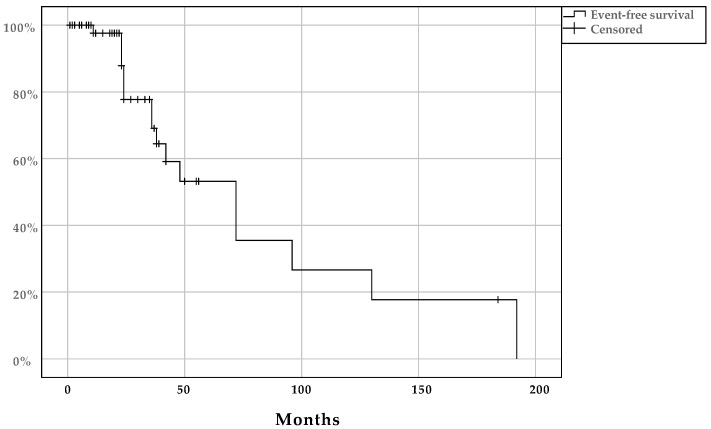
Kaplan–Meier plot with event-free survival in patients diagnosed with multiphenotypic sinonasal carcinoma (HMSC) within the head and neck region (including tonsils).

Patients diagnosed with HMSC within the head and neck region had 31.3 and 23 months mean and median follow-up time, respectively (Table 1). The outcome was defined in 59/78 cases (76%) and of these, 42/59 (71%) had NED at the end of their follow-up. Fifteen cases (25%) had an LR, 1/59 (2%) patients died from disease and another patient (1/59; 2%) had a DM. All patients’ event-free survival is presented in Figure 2. The mean and median event-free survival times were 81.7 months (95% CI: 52.1–111) and 72 months (95% CI: 41.1–103), respectively.

### 3.2. Histomorphology, Immunohistochemistry and HPV Status in HMSC

Tumors diagnosed as HMSC were often micro-morphologically described as a basaloid proliferation (78/79 cases; not defined in 1 case). The presence of solid areas was described in 76/79 cases (1 without solid areas and 2 not defined) and the presence of cribriform areas was observed in 53/79 cases (20 without cribriform areas and 6 not defined). Focal “squamous differentiation” within the tumor was observed in 15/79 cases (57 without squamous differentiation and 7 not defined). An associated dysplastic squamous epithelium was noticed in 50/79 cases (26 without and 3 not defined). Moreover, perineural (Pn) and lymphovascular invasion (LVI) were rarely observed and were detected in only 5 and 1 cases, respectively (Pn: 5/79 cases, 50 without and 24 not defined; LVI: 1/79 cases, 52 without and 26 not defined). A well-defined, at least focal, abluminal myoepithelial IHC staining (e.g., p40, p63, etc.) was, according to the authors of the manuscripts, observed in 53/79 cases (20 without and 6 not defined). At least focal luminal IHC staining (e.g., CD117) was identified in 40/70 cases (19 without and 20 not described).

Various methods were used to define HPV status and the most common method was the presence of p16 overexpression as a surrogate marker of the presence of HPV, used in 78/79 cases (78/78 cases within the head and neck region) (Table 1). HPV subtype was only assessed in a minority of cases, by PCR (*n* = 31). Here, HPV 33 dominated, with 17 cases followed by 4 cases with HPV35, 4 cases of HPV56 and 3 cases of HPV16 (Table 1); all types found were within species 9 of the alpha-papillomavirus genus [22].

## 4. Discussion

This is, to our knowledge, the first systematic review of HMSC in which all patients diagnosed with HMSC since the first report in 2013 were included and wherein we have attempted to summarize all available clinical and morphological data published so far. Taken together, the overall picture of the nature of the potentially new tumor entity HMSC has slightly changed since the initial summary reports [2,12,19,23], but is still mainly characterized by its indolent course despite an aggressive histomorphology.

Patients diagnosed with HMSC were clinically characterized by being young at diagnosis (53.7 years), without any sex predisposition and with a favorable prognosis after treatment, which most often consisted of surgery with/without RT. Interestingly, occurrence of distant metastasis was rare and no regional metastasis was reported and patients had a generally favorable survival rate. However, it is important to note that the follow-up time was lacking or was short in many cases and therefore the prognostic impact of the micromorphological diagnosis must be interpreted with caution.

The most common method to confirm the presence of HPV was to use the overexpression of p16 as a surrogate marker for HPV. In a minority of cases, the HPV subtype was determined by PCR. In these cases, HPV33 dominated, followed by HPV35, 56, 16, etc. Previous studies have emphasized the dominance of HPV33 as a feature of HMSC [2]. However, as mentioned, the number of cases with a defined HPV subtype was too limited to draw any clear conclusions about subtype dominance and all HPV types detected in HMSC are included in species 9 of the HPV alpha-genus [22]. Moreover, there is, to our knowledge, no evidence of tissue tropism nor morphological correlation for specific HPV types within the same species of HPV. Therefore, it is possible that the HPV type frequency may change as more cases as published and specific HPV types should not be considered a feature of any carcinoma type.

The histomorphology varied slightly between cases. Most cases were characterized by a basaloid proliferation with the presence of solid areas. Many cases also showed a tendency for a biphasic IHC staining. An obvious differential diagnosis to HMSC is adenoid cystic carcinoma (ACC), which could be ruled out in the majority of cases, e.g., by the presence of a dysplastic epithelium, “squamous differentiation”, absence of Pn and absence of MYB gene fusions (data not shown).

Another differential diagnosis, however, may be a basaloid squamous cell carcinoma (SCC), which is characterized by a basaloid atypical proliferation that may contain cribriform areas and glad-like spaces [24]. Notably, the classical basaloid SCC occurs mostly in men and is *rarely* observed in the nasal cavity, although it can be HPV-related; but in contrast to HMSC, it also often presents with nodal metastases [25].

Subsequently, although there seem to be clinical differences between a basaloid SCC and HMSC, their micromorphological distinctions are not always clear-cut. Moreover, this study also highlights that HMSC-like morphology may occur outside the sinonasal region and, therefore, further studies are needed to better discriminate between these tumor variants.

In addition, it is worth noting that no cases that arose within the sinonasal region presented with a regional metastasis; however, all cases disclosed outside that region did. Whether this finding is attributed to factors related to anatomical location or differences in tumor nature despite similar morphology remains to be elucidated.

In summary, HMSC is a new subtype, according to WHO 2017, of non-keratinizing squamous cell carcinoma of the sinonasal region with an indolent clinical course [26]. However, in our opinion, better clinical follow-up data and a more detailed tumor characterization are preferably needed before HMSC can finally be justified and better-defined as its own tumor entity.

## Figures and Tables

**Table 1:** Patients diagnosed with human papillomavirus multiphenotypic sinonasal carcinoma (HMSC) or tumors with HMSC phenotype outside the sinonasal region between 2013 and 2021 and their clinical and tumor characteristics.

Case	Age	Sex	Tumor Size (cm)	Site	Treatment	Outcome	Follow-Up (Months)	p16	HPV PCR	DNA ISH ^1^	RNA ISH	Ref
1	46	Female	4	nasal	Surgery + RT	NED	35	+	33	+(31/33)	+	[3]
2	63	Female	3	Maxillary/ethmoid	Surgery	NED	33	+	33	+(31/33)	+	[3]
3	48	Female	3	Ethmoid	Surgery	LR	24	+	35	+(16)	+	[3]
4	57	Male	3	Nasal, ethmoid/sphenoid	Surgery	NED	2	+	−	+(16)	+	[3]
5	61	Male	3	Nasal	Unknown	Unknown	Unknown	+	33	−(31/33)	+	[3]
6	51	Female	1	Nasal	Surgery + RT	NED	55	+	33	−(31/33)	+	[3]
7	73	Female	5	Nasal, maxillary, orbit	Surgery	NED	37	+	33	+(31/33)	+	[3]
8	40	Female	3	Nasal, ethmoid/sphenoid	CRT	LR	23	+	33	−(31/33)	+	[3]
9	35	Male	Unknown	Nasal	Unknown	Unknown	Unknown	+	ND	ND	+	[3]
10	38	Female	3.2	Nasal	Surgery + RT	LR	38	+	ND	ND	+	[3]
11	64	Male	Unknown	Maxillary	Surgery + RT	LR	48	+	ND	ND	+	[3]
12	72	Male	3.5	Nasal	Unknown	Unknown	Unknown	+	ND	ND	+	[3]
13	45	Male	8.5	Nasal, maxillary	Surgery + RT	NED	12	+	ND	ND	+	[3]
14	35	Female	4.3	Nasal, frontal, lacrimal duct	Surgery + RT	NED	23	+	ND	ND	+	[3]
15	49	Female	1	Nasal	Surgery	NED	19	+	ND	ND	+	[3]
16	58	Female	0.7	Nasal	Surgery + RT	LR	72	+	ND	ND	+	[3]
17	46	Female	3.6	Nasal	Surgery + RT	LR	24	+	ND	ND	+	[3]
18	36	Male	Unknown	Nasal	Unknown	Unknown	Unknown	+	ND	ND	+	[3]
19	28	Female	Unknown	Nasal	Unknown	LR	72	+	ND	ND	+	[3]
20	57	Female	3.9	Nasal, ethmoid, orbit	Surgery + RT	NED	12	+	ND	ND	+	[3]
21	76	Male	2.5	Nasal, orbit	Surgery + RT	NED	8	+	ND	ND	+	[3]
22	84	Female	6.1	Nasal, orbit, cranial fossa	Unknown	Unknown	Unknown	+	ND	ND	+	[3]
23	90	Male	4.5	Nasal	RT	Unknown	Unknown	+	ND	ND	+	[3]
24	65	Female	2.5	Nasal, maxillary	Surgery + RT	NED	21	+	ND	ND	+	[3]
25	46	Female	Unknown	Nasal	Unknown	LR	96	+	ND	ND	+	[3]
26	50	Male	7.2	Nasal, ethmoid	Surgery + CRT	NED	6	+	ND	ND	+	[3]
27	52	Female	Unknown	Nasal	Surgery + RT	LR	36	+	ND	ND	+	[3]
28	60	Female	3.9	Nasal	Surgery + RT	LR	130	+	56	+	+	[3]
29	53	Female	3	Nasal	Surgery	NED	56	+	33	+(33)	+	[3]
30	48	Female	4	Nasal	Surgery	NED	39	+	33	+(33)	+	[3]
31	51	Female	2	Nasal, ethmoid/sphenoid	Surgery + RT	NED	27	+	35	+	+	[3]
32	29	Male	5	Nasal	Surgery	LR	23	+	35	+	+	[3]
33	53	Male	3	Nasal	Surgery	NED	15	+	33	+	+	[3]
34	61	Male	7	Nasal	Surgery	NED	3	+	ND	ND	+	[3]
35	53	Male	Unknown	Nasal, frontal	Surgery	LR	36	+	ND	ND	+	[3]
36	51	Male	3.6	Nasal	Surgery + RT	LR	42	+	ND	ND	+	[3]
37	53	Male	5	Nasal	Surgery	NED	3	+	ND	ND	+	[3]
38	63	Female	6	Nasal, maxillary	Unknown	Unknown	Unknown	+	ND	ND	+	[3]
39	66	Male	3.7	Maxillary	Surgery	NED	3	+	ND	ND	+	[3]
40	50	Male	6.5	Nasal, maxillary/ethmoid	Surgery	NED	6	+	ND	ND	+	[3]
41	53	Female	3.8	Nasal	Surgery	NED	1	+	ND	ND	+	[3]
42	71	Female	Unknown	Ethmoid	Unknown	Unknown	Unknown	+	ND	ND	+	[3]
43	37	Female	3.5	Nasal, maxillary	Surgery + CRT	NED	42	+	ND	ND	+	[3]
44	51	Female	Unknown	Nasal	Unknown	Unknown	Unknown	+	ND	ND	+	[3]
45	48	Male	2.2	Nasal	Surgery	NED	22	+	ND	ND	+	[3]
46	58	Male	3,4	Nasal	Surgery	NED	3	+	ND	ND	+	[3]
47	46	Male	1.3	Nasal	Surgery	NED	8	+	ND	ND	+	[3]
48	46	Male	Unknown	Nasal	Unknown	Unknown	Unknown	+	ND	ND	+	[3]
49	60	Female	4.2	Nasal, maxillary	Surgery + CRT	NED	10	+	ND	ND	+	[3]
50	75	Female	2.5	Nasal	Surgery	NED	12	+	ND	+	ND	[5]
51	37	Male	3	Nasal	Surgery	Unknown	Unknown	+	*	+	ND	[6]
52	30	Male	0.9	Middle turbinate	Surgery	NED	184	+	*	+	ND	[6]
53	48	Male	2.2	Posterior nasal cavity	Surgery	NED	20	+	*	+	ND	[6]
54	46	Male	1.3	Middle turbinate	Surgery	NED	5	+	*	+	ND	[6]
55	58	Male	3.4	Middle turbinate	Surgery + RT	NED	3	+	*	+	ND	[6]
56	60	Male	3.5	Nasal, ethmoid	Surgery	Unknown	Unknown	+	35	ND	ND	[7]
57	46	Unknown	Unknown	Inferior turbinate	Surgery	Unknown	Unknown	+	33	ND	ND	[7]
58	69	Female	Unknown	Nasal	Surgery	LR	24	+	ND	ND(33)	+	[8]
59	42	Female	3	Nasal	Surgery	NED	Unknown	+	56, 68	ND	ND	[9]
60	60	Male	Unknown	Nasal	Surgery + RT	DOD	11	+	14	ND	ND	[10]
61	40	Male	1.6	Nasal	Unknown	Unknown	Unknown	+	ND	−	+	[11]
62	60	Male	1.5	Nasal	Unknown	Unknown	Unknown	+	ND	+	+	[11]
63	39	Female	3.5	Nasal	Unknown	Unknown	Unknown	+	ND	+	+	[11]
64	76	Male	1.5	Nasal	Unknown	Unknown	Unknown	+	ND	+	+	[11]
65	36	Female	1	Nasal	Unknown	Unknown	Unknown	+	ND	+	+	[11]
66	32	Female	2.4	Nasal	Unknown	Unknown	Unknown	+	ND	+	+	[11]
67	48	Female	9.5	Nasal	Surgery	NED	12	+	52	ND	ND	[12]
68	66	Male	Unknown	Nasal	Surgery	LR	192	+	33, 51	ND	ND	[13]
69	54	Female	Unknown	Nasal	Surgery	NED	9	+	16	ND	ND	[14]
70	70	Male	7.8	Nasal	Surgery + RT	NED	11	+	ND	ND	+	[15]
71	65	Female	Unknown	Middle concha	Surgery	NED	50	+	56	ND	ND	[16]
72	54	Female	Unknown	Nasal	Surgery	NED	38	+	82	ND	ND	[16]
73	46	Female	Unknown	Nasal	Surgery	NED	30	+	56	ND	ND	[16]
74	60	Female	Unknown	Nasal	Surgery + CRT	NED	3	+	33	ND	ND	[16]
75	65	Male	Unknown	Nasal	Surgery + RT	DM	23	+	16	ND	ND	[17]
76	49	Male	2	Nasal	Surgery	NED	18	+	33	ND	ND	[18]
77	84	Male	Unknown	Nasal	Surgery + RT	NED	6	+	ND	+(ND)	ND	[19]
78	54	Male	2	Tonsil	Surgery + RT	NED	24	+	ND	ND	+(16)	[20]
79	45	Female	10	Breast	Unknown	Unknown	Unknown	ND	ND	ND	+	[21]

Abbreviations: CRT: chemo-radiotherapy; DM: distant metastasis; DOD: dead of disease; LR: local relapse; ND: not determined; NED: no evidence of disease; RT: radiotherapy; +: positive; −: negative. * HPV subtype cannot be extracted case-wise from this study [6]; however, 4/5 cases were HPV33-positive and one cases was HPV16-positive. *^1^* categorized as X(Y), where X is wide probe set and Y is type-specific probe.

## Data Availability

Not applicable.
